# Attention and motor deficits index non-specific background liabilities that predict autism recurrence in siblings

**DOI:** 10.1186/s11689-017-9212-y

**Published:** 2017-09-05

**Authors:** Sabine E. Mous, Allan Jiang, Arpana Agrawal, John N. Constantino

**Affiliations:** 1 Department of Child and Adolescent Psychiatry/Psychology, Sophia Children’s Hospital, Erasmus Medical Center, Wytemaweg 80, 3015 CN Rotterdam, The Netherlands; 2 Division of Child Psychiatry, Department of Psychiatry, Washington University School of Medicine, 660 South Euclid Avenue, Campus Box 8504, St Louis, MO 63110 USA

**Keywords:** Autism, ADHD, Motor coordination, Sibling recurrence, Family studies

## Abstract

**Background:**

Recent research has demonstrated that subclinical autistic traits of parents amplify the effects of deleterious mutations in the causation of autism spectrum disorder (ASD) in their offspring. Here, we examined the extent to which two neurodevelopmental traits that are non-specific to ASD—inattention/hyperactivity and motor coordination—might contribute to ASD recurrence in siblings of ASD probands.

**Methods:**

Data from a quantitative trait study of 114 ASD probands and their brothers, 26% of whom also had ASD, were analyzed. Autistic trait severity was ascertained using the Social Responsiveness Scale-2, attention/hyperactivity problems using the Achenbach System of Empirically Based Assessment, and motor coordination (in a subset of participants) using the Developmental Coordination Disorder Questionnaire.

**Results:**

Among siblings (affected and unaffected), both categorical recurrence of ASD (Nagelkerke *R* ^2^ = 0.53) and quantitative ASD trait burden (*R* ^2^ = 0.55) were predicted by sibling ADHD and motor coordination impairment scores, even though these traits, on average, were not elevated among the unaffected siblings.

**Conclusions:**

These findings in a clinical family cohort confirm observations from general population studies that inattention/hyperactivity and motor impairment—axes of behavioral development that are non-specific to ASD, and often appreciable before ASD is typically diagnosed—jointly account for over 50% of the variation in autistic impairment of siblings, whether ascertained quantitatively or categorically. This finding within a sibling design suggests that background ASD susceptibilities that are inherited but non-specific (“BASINS”) may contribute to additive genetic liability in the same manner that ASD-specific susceptibilities (such as parental subclinical ASD traits and deleterious mutations) engender ASD risk.

**Electronic supplementary material:**

The online version of this article (doi:10.1186/s11689-017-9212-y) contains supplementary material, which is available to authorized users.

## Background

Autism spectrum disorder (ASD) is characterized by difficulties in social communication and restricted interests or repetitive behaviors. Recent epidemiologic research demonstrated that ASD traits are continuously distributed in the general population [1, 2] and are highly heritable; clinical autistic syndromes may arise from extreme aggregations of such continuously distributed traits or from the highly deleterious effects of genetic variants as occur in monogenic or oligogenic ASD syndromes [3]. Recently, significant advances in scientific understanding of the nature of the influence of common variation were made on the basis of two strategic trans-generational family studies. The first demonstrated that in the setting of de novo mutations conferring ASD risk (patients with de novo 16p11.2 deletions and their first degree relatives), the level of impairment of the individual with the mutation was significantly amplified if the *genetic background* as indexed by the bi-parental mean for subclinical autistic traits was in the *upper range of normal* [4]. The second, in a large epidemiologic sample, demonstrated that when both parents exhibited autistic trait aggregation in the upper quintile of the *normal distribution*, the risk for clinical-level ASD affectation of offspring was doubled [5].

Many previous studies have demonstrated the aggregation of *non-ASD-specific* neurodevelopmental impairments (e.g. motor or attentional impairment) in individuals affected by ASD, even though these inherited neurodevelopmental problems are not included in the DSM5 diagnostic criteria for autism spectrum disorder [6–8]. As yet, the nature and direction-of-effect of the genetic and developmental overlap between ASD and ADHD or between ASD and motor impairment remain incompletely specified. Although family and twin studies strongly suggest shared additive genetic liabilities between ASD and ADHD, findings from candidate gene, linkage, and genome-wide association studies are mixed [9]. Supporting causal overlap, molecular genetic studies have revealed highly pleiotropic effects of rare deleterious mutations—for example, those involving FMR1, TSC1/TSC2, NF1, and the 22q11 deletion—which have been variously associated with ADHD or ASD across individual carriers [10–12]. Furthermore, a large population-based twin study indicated that a substantial proportion of the genetic susceptibility for ASD symptomatology is shared with that for ADHD symptomatology, with almost 60% of the genetic influences shared by both disorders [13]. Tempering the notion of causal overlap, however, a recent genome-wide association study, using large case–control ASD and ADHD samples, did *not* identify significant overlap in common variant liability [14]. Here, it is important to recognize that very few common variants in this study were *individually* associated with either disorder at a level reaching genome-wide significance. Shared familial transmission of ASD and ADHD has been suggested in a study showing that mothers with an ADHD diagnosis did not only have an increased risk of having a child with ADHD but also had a 2.5-fold increased risk of having a child with ASD [15]. Furthermore, studies suggest that siblings of children with ASD not only have a 20-fold relative risk of also developing ASD [16] but also have an increased risk of developing ADHD. In a recently published study of such co-aggregation, it was shown that 5.3% of the ASD-affected probands had at least one sibling with an ADHD diagnosis, versus 1.5% of the non-ASD-affected probands (adjusted risk ratio 3.7) [17]. Another study showed that the prevalence of an ADHD diagnosis was 15% among dizygotic co-twins of children with ASD [13], while the most recent US estimates place the general population prevalence of ADHD between 5 and 8%. Similarly, higher ASD symptom levels have been reported in siblings of children with ADHD [18].

Regarding motor coordination, a majority of children with ASD manifest some degree of impairment [19], the severity of which has been found to be strongly correlated with the degree of social communication impairment [8, 20, 21]. This is extremely important, because the measurement of motor impairment is far less likely to be confounded with measurement of social impairment than might be the case for other behavioral comorbidities. To this effect, shared additive genetic influences of ASD and developmental coordination disorder (DCD) have been demonstrated previously, showing that ASD and DCD shared about 40% of their respective genetic influences and that about 30% of children with an ASD diagnosis also met the criteria for a DCD diagnosis [13]. Similarly, a clear overlap between ADHD and DCD was found; among children with ADHD, the prevalence of DCD was about 10 times higher compared to the general population, and a similarly heightened prevalence of ADHD was reported in children with DCD [13]. Other genetically informative studies have demonstrated that both ADHD symptoms and motor problems are highly heritable [22].

Finally, a separate body of research has explored neurodevelopmental correlates of the co-occurrence of ADHD symptoms and motor impairments, beginning with specific groups of clinically ascertained patients for whom the term “DAMP syndrome” (deficits in attention, motor proficiency, and perception) was coined [23]. These patients were found to have substantially higher frequencies of clinical-level autistic symptomatology compared to children in the general population or children with ADHD symptoms only [23, 24]. When these associations were tested within an epidemiologic sample (in a study that actually excluded patients with ASD), Reiersen and colleagues similarly demonstrated that co-occurrence of attention problems and motor coordination impairments was associated with substantially elevated autistic trait burden [25].

In this study, we capitalized upon a sibling design to conduct a first ever analysis determining whether ADHD symptoms and impairments in motor coordination, which are non-specific to ASD, nevertheless represent a source of additive genetic background liability for ASD, when in the presence of inherited ASD-specific risk conferred by the status of being a later-born sibling of an ASD-affected proband. To our knowledge, no prior study has examined the extent to which quantitative variation in these traits among siblings in ASD-affected families contributes to ASD recurrence.

## Methods

### Sample

In total, there were 307 males in the original study sample. In families with multiple individuals diagnosed with ASD, proband status was assigned to the older affected brother. Monozygotic twins of probands, individuals with trisomy-21 and Fragile X syndrome, individuals without data, and individuals falling outside of age limits of instruments were excluded. For families with multiple male siblings of an affected proband, selection of the sibling was based on the amount of data available, the sibling closest-in-age to the proband, or using a random number generator. The final number of children included in the study was 228: 114 males with a clinical diagnosis of ASD and 114 of their male siblings (one per family). All were participants in a study of quantitative autistic traits over the life course [26]. The probands were recruited by their physicians (between 2003 and 2005) from either (a) the Washington University Child and Adolescent clinics or (b) from outpatient child psychiatry practices in the greater St. Louis metropolitan area. Any child with an ASD diagnosis documented by an expert clinician and who had at least one brother was eligible for inclusion. Diagnostic status of affected children was confirmed using the Autism Diagnostic Interview-Revised (ADI-R) [27]. The mean age at enrollment was 6.9 years (range 3–16 years) for the brothers and 7.1 years (range 3–18) for probands. The sample was 89% Caucasian, 4% Hispanic, 4% Asian, 2% African-American, and 1% other or bi-racial. Of the 114 siblings, 26% (*n* = 29) also had an ASD diagnosis and 94% (*n* = 107) were verbal.

### Measures

#### Autistic traits

Autistic traits were assessed using the Social Responsiveness Scale-2 (SRS-2) [28] by parent and teacher report. The SRS-2 is a 65-item measure of quantitative autistic traits (QAT), using a 4-point Likert scale (not true, sometimes true, often true, almost always true) for each item. The SRS-2 items encompass both of the DSM-5 criterion domains for ASD (social communication/interaction and restricted/repetitive patterns of behavior, interests, or activities). Scores on the SRS-2 have been found to be highly heritable [29–31], extremely stable over time [26], continuously distributed in the general population [30], exhibit a unitary factor structure [32], and distinguish children with autism spectrum conditions from those with other child psychiatric conditions [33]. The SRS-2 yields a total problem score, which has been empirically validated by factor, cluster, and latent class analyses [32]. In this study, raw SRS-2 total scores were converted to standardized *T* scores (mean 50, SD 10), where higher scores indicate greater impairment. The total score is truncated at the low end of the scales, so that a *T* score of 30 is the minimum obtainable. A total *T* score of 76 or higher is consistent with severe clinical-level symptomatology, a *T* score of 60 through 75 subclinical, and a *T* score of 59 or less as normal.

The SRS-2 was completed by 113 parents of siblings, 107 parents of probands, 107 teachers of siblings, and 107 teachers of probands. Raw scores were converted to *T* scores (mean 50, SD 10). Higher scores indicate greater impairment.

#### Attention-deficit/hyperactivity symptoms

Attention-deficit/hyperactivity symptoms were assessed by parent and teacher report, using the Achenbach System of Empirically Based Assessment (ASEBA) [34, 35]. In the Child Behavior Checklist (CBCL) and Teacher Report Form (TRF), the primary caregiver and teacher are asked to report on the behavior of the child in the preceding months, using a 3-point Likert scale (not true, somewhat or sometimes true, and very true or often true) for each item. The CBCL and TRF 1.5–5 consist of 99 items, the CBCL 6–18 of 113 items, and the TRF 6–18 of 115 items. All versions yield a total problem score as well as scores on syndrome scales and DSM-oriented scales. In this study, the extensively validated DSM-oriented Attention-Deficit/Hyperactivity Problems (ADHP) Scale score was used. Raw scores were converted to standardized *T* scores (mean 50, SD 10). Higher *T* scores indicate greater impairment. The problem score is truncated at the low end of the scale, so that a *T* score of 50 is the minimum obtainable. A *T* score between 65 and 70 on the DSM-oriented scales is considered borderline clinical and a score above 70 as clinical.

In total, 114 parents completed the ASEBA CBCL in siblings and probands. The ASEBA TRF was completed by 106 teachers of siblings and 113 teachers of probands.

#### Motor proficiency

The Developmental Coordination Disorder Questionnaire (DCDQ’07; revised 2007 edition) was completed by parents on a subset of siblings (*n* = 39) and probands (*n* = 44). Data was only available in a subset of participants because the collection of DCDQ data was added to the study protocol after a first wave of patients had already completed their follow-up. The DCDQ is a 15-item questionnaire that ascertains gross and fine motor skill impairments that would contribute to a diagnosis of DCD [36]. Moderate correlations have been found between DCDQ scores and other measures of motor proficiency and visual motor integration (Movement Assessment Battery for Children; *r* = 0.55) [37] and the Beery Test of Visual-Motor Integration (*r* = .42) [38], supporting the construct (convergent) validity of the DCDQ. A previous study has also shown a strong correlation (*r* = 0.79) between total DCDQ and total Bruininks-Oseretsky Test of Motor Proficiency, Second Edition (BOT-2) scores in families with ASD, suggesting that the DCDQ can be used as a reliable proxy for the measurement of motor impairment in this population [8]. High internal consistency and predictive, construct, and concurrent validity and good sensitivity and specificity have been reported for the DCDQ [36, 39]. The DCDQ yields a raw total score (score range 15–75) which was age-adjusted and incorporated into the analyses of this study; higher scores indicate better motor functioning.

### Data analysis

All analyses were performed using the IBM SPSS Statistics, version 20 [40].

For descriptive purposes, independent samples *t* tests were performed to compare trait distributions between affected and unaffected children, as well as between siblings and probands. To study the extent to which trait variation was associated within families, intraclass correlation coefficients (ICC; two-way mixed, absolute agreement, average measure) between siblings and probands in each pair were calculated. Furthermore, to show the relation between the various measures, non-parametric bivariate correlations were calculated in the siblings. Finally, since DCDQ data was only available in a subset of participants, we performed an independent samples *t* test comparing the individuals with and without available DCDQ data on the SRS QAT and CBCL/TRF ADHP scores to rule out potential selection bias.

Next, we performed statistical prediction models. First, we examined the extent to which *ASD diagnostic status* of siblings could be predicted exclusively by the level of their attention and motor problems; hierarchical binary logistic regression analyses were performed. Finally, to assess whether *quantitative variation in autistic trait severity* among siblings could be predicted by attention and motor problems, hierarchical linear regression analyses were performed. To isolate the effect of non-ASD-specific additive genetic background liability for ASD, both the logistic and linear statistical prediction models were corrected for severity of affectation of the proband (acting as a proxy for inherited ASD-specific risk).

## Results

### Descriptives

Table 1 shows the descriptives of the sample and provides a comparison between probands and (unaffected + affected) siblings. In supplemental Additional file 1: Table S1 (online), the descriptives are presented according to ASD affectation status, comparing probands, affected siblings, and unaffected siblings. The results in Additional file 1: Table S1 show that as expected, mean QAT scores were significantly higher in ASD-affected than in unaffected individuals, with an about 3 SD difference in mean scores as reported by parents, and a 2 SD difference in mean scores as reported by teachers (SRS *T* score SD = 10). Among ASD-affected individuals, the mean ADHP scores were about 0.8 SD higher, reported by both parents and teachers (CBCL/TRF *T* score SD = 10). Also, ASD-affected and ASD-unaffected individuals differed significantly on the DCDQ. We emphasize here that the mean ADHP and DCDQ scores for unaffected siblings were well within the normal range and in keeping with means for the general population [34–36].

**Table 1:** Descriptive statistics

		Siblings (unaffected + affected)		Probands		*t* (*p*)	Cohen’s *d* ^a^	ICC (*p*)^b^
		*n*	Mean (SD)	*n*	Mean (SD)			
SRS-2 score								
	Parent	113	53.1 (15.1)	107	75.7 (12.3)	12.1 (<0.001)	1.64	0.15 (0.027)
	Teacher	107	55.1 (15.0)	107	68.3 (10.8)	7.4 (<0.001)	1.01	0.32 (0.002)
CBCL/TRF ADHP score								
	Parent	114	55.9 (8.0)	114	62.0 (9.4)	5.3 (<0.001)	0.70	0.20 (0.071)
	Teacher	106	55.4 (7.6)	113	60.2 (7.7)	4.7 (<0.001)	0.63	0.13 (0.196)
DCDQ score								
	Parent	39	60.2 (15.9)	44	44.2 (14.0)	−4.9 (<0.001)	1.07	0.01 (0.486)
		*n* (%)		*n* (%)		*χ* ^2^ (*p*)		*Φ* ^a^
Clinical diagnosis						137.8 (<0.001)		0.78
	Autism	14 (12.3)		34 (29.8)				
	ASD	15 (13.1)		80 (70.2)				
	No ASD	85 (74.6)		0 (0.0)				
Expressive language						2.6 (0.104)		0.11
	Nonverbal	3 (2.6)		8 (7.0)				
	Verbal	107 (93.9)		97 (85.1)				
	Missing	4 (3.5)		9 (7.9)				
ADOS-2 classification								
	Autism	–		70 (61.4)				
	ASD	–		17 (14.9)				
	Non-spectrum	–		13 (11.4)				
	Missing	–		14 (12.3)				

For SRS-2, CBCL, and TRF, a higher score indicates more severe impairment. For DCDQ, a higher score represents better functioning. For the SRS-2, a *T* score of 30 is the minimum obtainable. A total *T* score of 76 or higher is consistent with severe clinical-level symptomatology, a *T* score of 60 through 75 subclinical, and a *T* score of 59 or less as normal. For the CBCL and TRF, a *T* score of 50 is the minimum obtainable. A *T* score between 65 and 70 is considered borderline clinical and a score above 70 as clinical

*SRS-2* Social Responsiveness Scale-2 (*T* score), *CBCL/TRF ADHP* DSM-oriented Attention-Deficit/Hyperactivity Problems Scale (*T* score), from Child Behavior Checklist and Teacher Report Form, *DCDQ* Developmental Disorder Coordination Questionnaire (adjusted total score), *ASD* autism spectrum disorder

^a^Effect sizes reported as Cohen’s *d* for *t* tests and phi (*φ*) for chi-square tests, with 0.1 considered a small effect, 0.3 a medium effect, and 0.5 or higher a large effect

^b^Intraclass correlation coefficients (ICC; two-way mixed, absolute agreement, average measure) are provided, calculated between siblings and probands in each pair, depicting variation within families

To study the extent to which variation in the respective traits was associated within families, intra-class correlation coefficients (ICC) were calculated (Table 1). We observed sibling correlations (ICC) on the order of 0.13–0.32 for QAT and ADHP, consistent with established heritability estimates for each of these parameters of child development and the expected attenuation of such correlations when both clinically affected and non-affected family members are included. In supplemental Additional file 1: Table S2 (online), ICC values are depicted for ASD concordant and discordant sibling pairs separately. As expected, ICC values are significantly larger in concordant sibling pairs.

Finally, an independent samples *t* test was performed, comparing the individuals with and without available DCDQ data on the SRS QAT and CBCL/TRF ADHP scores to rule out selection bias. We found no significant differences in autistic trait severity (*t* (218) = 0.25 and *p* = 0.800 and *t* (212) = −1.25 and *p* = 0.212 for parent and teacher report, respectively) or ADHD severity (*t* (226) = 0.17 and *p* = 0.867 and *t* (217) = −1.03 and *p* = 0.306 for parent and teacher report, respectively) between participants with or without DCDQ data.

**Fig. 1:**
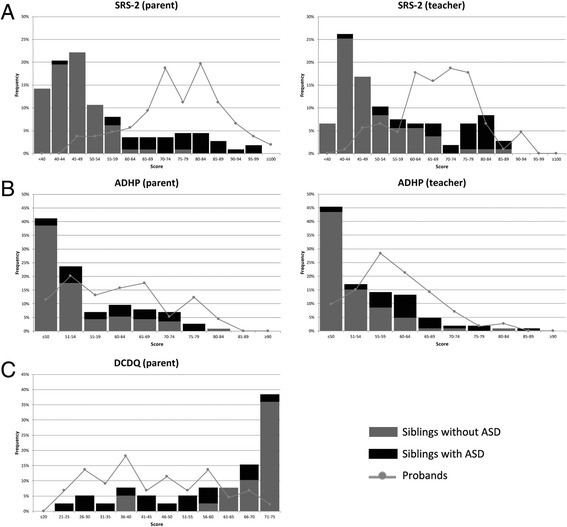
Score distributions of siblings and probands. **a** SRS-2 *T* scores. **b** CBCL/TRF ADHP *T* scores. **c** DCDQ age-adjusted scores. For SRS-2, CBCL, and TRF, a higher score indicates more severe impairment. For DCDQ, a higher score represents better functioning. For the SRS-2, a *T* score of 30 is the minimum obtainable. A total *T* score of 76 or higher is consistent with severe clinical-level symptomatology, a *T* score of 60 through 75 subclinical, and a *T* score of 59 or less as normal. For the CBCL and TRF, a *T* score of 50 is the minimum obtainable. A *T* score between 65 and 70 is considered borderline clinical and a score above 70 as clinical. *SRS-2* Social Responsiveness Scale-2, *CBCL/TRF ADHP* DSM-oriented Attention-Deficit/Hyperactivity Problems Scale, from Child Behavior Checklist/Teacher Report Form, *DCDQ* Developmental Disorder Coordination Questionnaire

Figure 1 displays the trait distributions of the three different measures. Scores were continuously distributed for both probands and siblings for all measures, with substantial floor effects for ADHP scores, as expected from rating systems in which *T* scores are truncated. Similarly, DCDQ motor proficiency scores exhibited a ceiling effect among unaffected siblings. Substantial pathological shifts in the distribution of ADHD and motor proficiency scores were appreciable for the probands, as well as for ASD-affected siblings, but not for unaffected siblings.

Bivariate (Spearman) correlations were calculated (Table 2). Correlations are presented here exclusively for the siblings as a group (affected and unaffected), because these variables are being explored to predict recurrence and to offer the widest and most representative distribution of scores in which to examine trait correlations.

**Table 2:** Bivariate non-parametric (Spearman) correlations

	SRS-2 (parent)		SRS-2 (teacher)		CBCL ADHP (parent)		TRF ADHP (teacher)	
	*r*	*n*	*r*	*n*	*r*	*n*	*r*	*n*
SRS-2 (teacher)	0.70**	106						
CBCL ADHP (parent)	0.58**	113	0.42**	107				
TRF ADHP (teacher)	0.54**	105	0.70**	102	0.49**	106		
DCDQ (parent)	−0.65**	38	−0.56**	36	−0.33*	39	−0.54**	37

Note: siblings only. For SRS-2, CBCL, and TRF, a higher score indicates more severe impairment. For DCDQ, a higher score represents better functioning

*SRS-2* Social Responsiveness Scale-2 (*T* score), *CBCL/TRF ADHP* DSM-oriented Attention-Deficit/Hyperactivity Problems Scale (*T* score), from Child Behavior Checklist and Teacher Report Form, *DCDQ* Developmental Disorder Coordination Questionnaire (adjusted total score)

*p < 0.05; **p < 0.01

A strong positive correlation was found between the teacher-reported SRS-2 autistic trait score and that of parent-reported SRS-2 autistic traits (*r* = 0.70), indicating high cross-informant validity of the scores in this sample of affected and unaffected children. A concomitantly low level of parent-teacher agreement on ADHD traits and the general superiority of teacher ratings for these symptoms motivated prioritization of teacher-report ADHD ratings in our prediction model. Differentiating the source of information for prediction (teachers) versus outcome (parents, clinicians) for the respective behavioral indices of ADHP and QAT minimized effects of rater bias and optimized validity and interpretation of regression analysis results.

### Attention-deficit/hyperactivity and motor traits predicting ASD diagnosis

Hierarchical binary logistic regression analyses were performed to examine the extent to which a categorical ASD diagnosis in a sibling could be predicted by teacher-reported ADHD symptom scores and parent-reported motor proficiency scores, controlling for proband ASD severity (Table 3).

**Table 3:** Logistic regression analyses predicting sibling diagnosis

	Model 1		Model 2a		Model 2b		Model 3		Model 4		Model 5	
	*n* = 35		*n* = 35		*n* = 35		*n* = 35		*n* = 35		*n* = 35	
	OR (95% CI)	*p*	OR (95% CI)	*p*	OR (95% CI)	*p*	OR (95% CI)	*p*	OR (95% CI)	*p*	OR (95% CI)	*p*
Proband SRS-2 score (teacher report)	1.02 (0.97–1.08)	0.461	1.02 (0.95–1.08)	0.609	1.01 (0.95–1.09)	0.729	1.02 (0.94–1.09)	0.678	1.02 (0.94–1.09)	0.678		
Sibling TRF ADHP score (teacher report)			1.15 (1.01–1.30)	0.033			1.09 (0.97–1.23)	0.144	1.09 (0.97–1.23)	0.159	1.09 (0.97–1.22)	0.155
Sibling DCDQ score (parent report)					0.91 (0.85–0.97)	0.003	0.92 (0.86–0.98)	0.010	0.92 (0.86–0.98)	0.013	0.92 (0.86–0.98)	0.009
TRF ADHP × DCDQ interaction									1.00 (0.98–1.01)	0.640		
Nagelkerke *R* ^2^	0.022		0.283		0.464		0.531		0.537		0.527	

Note: *n* = 35 for all models (only siblings with all data available were included). For SRS-2 and TRF, a higher score indicates more severe impairment. For DCDQ, a higher score represents better functioning

SRS-2 Social Responsiveness Scale-2 (*T* score), *TRF ADHP* DSM-oriented Attention-Deficit/Hyperactivity Problems Scale (*T* score), from Teacher Report Form, *DCDQ* Developmental Disorder Coordination Questionnaire (adjusted total score)

Proband SRS-2 autistic trait score alone (model 1) did not predict sibling ASD diagnosis (*χ* ^2^ (1) = 0.556, *p* = 0.456, Nagelkerke *R* ^2^ = 0.022). The model improved dramatically when sibling ADHP score was added (model 2a), resulting in significant overall fit of the model (*χ* ^2^ (2) = 8.013, *p* = 0.018, Nagelkerke *R* ^2^ = 0.283) and an overall correctly classified percentage of 80%. The TRF ADHP score was an important and highly significant predictor of ASD status (OR = 1.15, *p* = 0.033), showing that for each unit increase in TRF ADHP *T* score, the odds of an ASD diagnosis was increased by 15%. When sibling DCDQ motor proficiency score was added first to proband autistic trait score (model 2b), the model also improved, resulting in significant overall fit of the model (*χ* ^2^ (2) = 14.308, *p =* 0.001, Nagelkerke *R* ^2^ = 0.464) and an overall correctly classified percentage of 80%. The DCDQ score was an important and highly significant predictor of ASD status (OR = 0.91, *p* = 0.003), showing that for each unit increase in DCDQ motor proficiency score, the odds of an ASD diagnosis was decreased by 9%. When both sibling TRF ADHP and DCDQ motor proficiency scores were added (model 3), the model fit increased further (*χ* ^2^ (3) = 16.963, *p* = 0.001, Nagelkerke *R* ^2^ = 0.531) and an overall correctly classified percentage of 77% was achieved using these three variables. In this model, the DCDQ score (OR = 0.92, *p* = 0.010) was the most important predictor of ASD diagnostic status. To study potential interaction effects of the TRF ADHP score and the DCDQ score, analyses were repeated while adding an interaction term (model 4). Results show that the interaction term was insignificant and the model fit did not further improve (*χ* ^2^ (4) = 17.223, *p* = 0.002, Nagelkerke *R* ^2^ = 0.537). Finally, the analyses were repeated without the correction for proband ASD affectation severity (model 5), showing the minimal contribution of this variable to the model as compared to model 3.

Supplemental analyses were performed including both teacher- and parent-reported ADHD measures. The fit of the complete model (including proband autistic trait severity, teacher- and parent-reported ADHD scores, and the motor proficiency score) was good (*χ* ^2^ (4) = 25.841, *p* < 0.001, Nagelkerke *R* ^2^ = 0.722), with an overall correctly classified percentage of 89% (Additional file 1: Table S3, online). Additional analyses with reversed reporters showed similar results to the original analyses (Additional file 1: Table S4, online). Also, similar results were found when analyses were repeated in the entire sample, thus not constrained to the set of participants with complete data (Additional file 1: Table S5, online). Finally, to study the specificity of our findings, analyses were repeated with the other available TRF DSM-oriented scales. The results showed that affective and oppositional defiant problems were also predictive of ASD diagnostic status, although less strong than ADHD problems (Additional file 1: Table S6, online).

### Attention-deficit/hyperactivity and motor traits predicting autistic trait severity

Next, we examined the extent to which ADHP and DCDQ scores predicted quantitative autistic trait ratings among siblings of probands. We implemented hierarchical linear regression analyses in which sibling SRS-2 autistic trait severity (as reported by parents) was predicted by teacher-reported ADHP scores and parent-reported DCDQ motor proficiency score, controlling for teacher-reported proband SRS-2 autistic trait score (Table 4).

**Table 4:** Linear regression analyses predicting parent-reported autistic trait severity in siblings

	Model 1		Model 2a		Model 2b		Model 3		Model 4		Model 5	
	*n* = 35		*n* = 35		*n* = 35		*n* = 35		*n* = 35		*n* = 35	
	*β*	*p*	*β*	*p*	*β*	*p*	*β*	*p*	*β*	*p*	*β*	*p*
Proband SRS-2 score (teacher report)	0.30	0.086	0.26	0.098	0.26	0.098	0.19	0.109	0.19	0.111		
Sibling TRF ADHP score (teacher report)			0.45	0.005			0.24	0.066	0.25	0.074	0.24	0.063
Sibling DCDQ score (parent report)					−0.68	<0.001	−0.60	<0.001	−0.60	<0.001	−0.62	<0.001
TRF ADHP × DCDQ interaction									0.03	0.793		
Adjusted *R* ^2^	0.059		0.247		0.517		0.554		0.540		0.530	

Note: *n* = 35 for all models (only siblings with all data available were included). For SRS-2 and TRF, a higher score indicates more severe impairment. For DCDQ, a higher score represents better functioning

*SRS-2* Social Responsiveness Scale-2 (*T* score), *TRF ADHP* DSM-oriented Attention-Deficit/Hyperactivity Problems Scale (*T* score), from Teacher Report Form, *DCDQ* Developmental Disorder Coordination Questionnaire (adjusted total score)

Again, proband SRS-2 autistic trait score alone (model 1) minimally accounted for sibling SRS-2 autistic trait severity (*F* (1.33) = 3.137, *p* = 0.086). When sibling TRF ADHP score was added to the model (model 2a), the model fit improved substantially (model 1 to 2a Δ*R* ^2^ = 0.204, *p* = 0.005). The overall model was significant (*F* (2.32) = 6575, *p* = 0.004) and explained 25% of the variance in SRS-2 autistic trait severity. In this model, the TRF ADHP score was a very strong predictor (*β* = 0.45, *p* = 0.005). When sibling DCDQ motor proficiency score was added first to proband SRS-2 autistic trait score (model 2b), the model fit (*F* (2.32) = 19.200, *p* < 0.001) also improved significantly (models 1 to 2b Δ*R* ^2^ = 0.459, *p* < 0.001), explaining 52% of the variance in sibling SRS-2 autistic trait severity. When both sibling TRF ADHP score and DCDQ score were in the model (model 3), it appeared that adding the DCDQ score to the ADHP score significantly improved the model (models 2a to 3 Δ*R* ^2^ = 0.302, *p* < 0.001), while adding the ADHP score to the DCDQ score only marginally improved the model (models 2b to 3 Δ*R* ^2^ = 0.048, *p* = 0.066). The overall model fit including both sibling TRF ADHP score and DCDQ score was excellent (*F* (3.31) = 15.073, *p* < 0.001), and the proportion of explained variance showed that proband SRS-2 autistic trait score, sibling TRF ADHP score, and sibling DCDQ motor proficiency score jointly explained 55% of the variance in sibling SRS-2 autistic trait score. Again, the strongest predictor in this model was the DCDQ motor proficiency score (*β* = −0.60, *p* < 0.001). As in the logistic regression analyses, we also tested a model including the ADHP-by-DCDQ interaction term (model 4). Results show that the interaction term was insignificant and that the model fit decreased (*F* (4.30) = 10.983, *p* < 0.001, and models 3 to 4 Δ*R* ^2^ = 0.001, *p* = 0.793). A final analysis without correction for proband ASD affectation severity (model 5) again showed the minimal contribution of this variable to the model as compared to model 3.

Again, supplemental analyses were performed. First, analyses were repeated while both including teacher- and parent-reported ADHD measures, showing that up to 71% of the variance in autistic trait severity could be explained (Additional file 1: Table S7, online). Results remained similar to the original analyses when reversed reporters were used (Additional file 1: Table S8, online). When analyses were repeated in the entire sample, thus not constrained to the set of participants with complete data, all results remained identical (Additional file 1: Table S9, online). Also, the analyses were repeated exclusively predicting autistic trait severity in the unaffected siblings, and similar (but somewhat attenuated) results were found (Additional file 1: Table S10, online). Finally, specificity was studied, showing that affective and oppositional defiant problems were also predictive of autistic trait severity, although less strong than ADHD problems (Additional file 1: Table S11, online).

## Discussion

In this contemporaneous analysis of symptom burden of non-ASD-specific neurodevelopmental traits and ASD recurrence in the siblings of affected probands, we observed that ADHD symptoms and motor coordination impairment jointly accounted for a large share of the variance (over 50%) in both categorical ASD recurrence and quantitative trait severity. These findings in a clinical family cohort confirm observations from general population studies indicating that inattention/hyperactivity and motor coordination impairment—axes of behavioral development that exhibit trait-like stability, have been shown to be correlates of ASD symptomatology [41–45] and which were, *on average*, normal in the sibling group—account for approximately half of the variation in ASD recurrence, whether ascertained quantitatively or categorically, and controlling for the degree of ASD-specific background genetic liability indexed by the severity of affectation of the proband. Coupled with the observation of a lack of interaction effects between ADHD and motor coordination impairment, these findings suggest that ADHD and motor coordination impairments constitute contributors of additive risk for ASD, with motor coordination impairments adding the larger share.

**Fig. 2:**
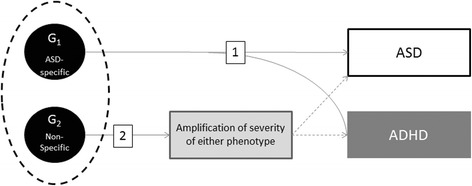
Mechanisms by which genetic influences that are non-specific to autism may compound autistic severity *and* incur “comorbid” affectation with non-ASD traits (ADHD as an example here). *1* Specific influences on ASD that simultaneously incur comorbidity traits that are part and parcel of the syndrome. *2* Amplification of ASD severity by a non-ASD-specific causal influence. *ADHD* attention-deficit/hyperactivity disorder, *ASD* autism spectrum disorder

This finding within a sibling design suggests that background ASD susceptibilities that are inherited but non-specific (“BASINS”) may contribute additive genetic liability for autism in the same manner that ASD-specific susceptibilities (such as parental subclinical ASD traits and deleterious mutations) engender ASD risk. In this way, non-specific (genetic and environmental) influences may amplify the effect of ASD-specific susceptibility on autism severity (Fig. 2).

This particular role in contributing non-ASD-specific risk could potentially explain elements of “missing heritability” for autism and may help resolve *apparent* discrepancies between genetic epidemiologic (population-based) and molecular genetic (case–control) studies in estimating the extent of genetic overlap between autism and ADHD, since the latter depend upon disease-specific associations that reach a critical threshold of statistical significance and generally do not control for subclinical cross-trait aggregation among controls—the latter should be strongly considered for inclusion in future molecular genetic case–control studies.

It should be noted that BASINS are most likely not restricted to ADHD and motor impairments only; other non-ASD-specific phenotypes have also been associated with ASD diagnostic status and autistic trait severity. Furthermore, it should be noted that clinical scores on these non-ASD-specific measures may also arise from reciprocal influences of core ASD symptomatology on these traits over the course of early childhood development.

Beyond the issue of genetic overlap, these findings have important implications for the phenomenology of infant development and the clarification of early liabilities that might contribute to the development of autism. In recent studies of the early development of ASD among high-risk infant siblings of children with ASD, trajectories of delayed motor development have been shown to predict later ASD diagnosis [41–43]. Similarly, early abnormalities in visual social engagement predict ASD among high-risk infant siblings [44, 45]. Given the high prevalence of motor impairments and attention/hyperactivity problems in ASD, either or both might serve as important early targets for intervention, both with respect to reducing non-specific neurodevelopmental liabilities that may directly contribute to the syndrome (ASD) and to reducing so-called comorbidity in affected children. For example, studies have shown that developmental therapies targeted to the acquisition of motor skills may have broad-ranging positive effects on the development of executive functioning, reduce symptoms of inattention/hyperactivity, and improve social behavior [46, 47]. These and other findings strongly reinforce the clinical implication that children with any significant degree of ASD symptom burden or risk should be screened systematically for motor impairment and attention problems at the earliest juncture at which the respective conditions might be safely intervened and improved.

It remains unclear—and a potential clue to tracing the neural underpinnings of ASD—why the capacity for reciprocal social communication would track so closely with motor coordination and with variation in attention/hyperactivity. Although the timing of motor and attentional abnormalities makes it possible that they precede and contribute to the development of autistic symptomatology, the direction of effect between autism and these comorbidities cannot by any means be resolved by this study design, and this—along with the restriction of the available sample to males only—represents the most significant limitation of this study. Previous studies have construed motor impairment and ADHD symptomatology as “secondary comorbidities” to clinical ASD, but it is possible that ASD arises secondarily as a specific type of complication, decompensation, or epiphenomenon when a critical additive mass of neurodevelopmental liability compromises social maturation [48]. The fact that ADHD and motor coordination problems did not preferentially aggregate in the unaffected siblings of our ASD probands (yet robustly predicted categorical and quantitative recurrence) raises the possibility that either (a) the direction of causation is actually from these secondary traits to ASD rather than the other way around or (b) a more fundamental developmental abnormality is responsible for the emergence of all three sets of correlated symptoms.

A further limitation of this study is that motor proficiency data were only available in a subset of participants, potentially attenuating the estimation of the true effect. Future, larger, prospective studies should examine these associations in a developmental context, which would allow for direction of causation to be more directly tested. Furthermore, our measures of ADHD symptoms and QAT were conducted by multi-informant questionnaire ratings rather than direct observation; we note however that the instruments used have been extensively validated and normed among many thousands of individuals in the general population [34, 35, 49, 50]. The use of independent informants minimized the effect of rater bias and optimized validity and interpretation of the results. Finally, although systematic measurements of IQ were not conducted in this study, only verbal, non-intellectually disabled siblings were eligible, and it has been well established that variation in QAT using the methods implemented in this study are unrelated to IQ within the normal range of variation in cognition in the general population [51].

Based on these and the previous findings, it will be important for the current generation of prospective studies of infant siblings of children with ASD to incorporate the evaluation of motor functioning and ADHD symptoms in longitudinal data collection. Relating early abnormalities in these functions to brain development, neurotransmitter systems, genetic variants, and other biomarkers may lend key insights into the biology of ASD.

## Conclusions

To conclude, our findings in a clinical family cohort confirm observations from general population studies that inattention/hyperactivity and motor impairment—axes of behavioral development that are non-specific to ASD, and often appreciable before ASD is typically diagnosed—jointly account for over 50% of the variation in autistic impairment of siblings, whether ascertained quantitatively or categorically. This suggests that BASINS may contribute to additive genetic liability in the same manner that ASD-specific susceptibilities (such as parental subclinical ASD traits and deleterious mutations) engender ASD risk. Future biomarker and molecular genetic studies should strongly consider cross-trait ascertainment, particularly among controls, as a means of capturing “missing heritability” that might be tagged to genetic factors that are non-specific to ASD. Early interventions capable of improving or resolving early non-specific developmental liabilities that may contribute risk for ASD can be directly tested for their ability to ameliorate ASD severity, particularly among infants known to be at elevated risk.

## Additional file

Additional file 1: Supplemental results. Description of data: Tables with results of additional (supplementary) analyses are provided. (DOCX 64 kb)
