# Supplementary material for: Attention and motor deficits index non-specific background liabilities that predict autism recurrence in siblings
Source: J Neurodev Disord. 2017 Sep 5;9:32. doi: 10.1186/s11689-017-9212-y (PMC5583755; doi:10.1186/s11689-017-9212-y)
Supplement: Additional file 1: — Supplemental results. Description of data: Tables with results of additional (supplementary) analyses are provided. (DOCX 64 kb) [file 11689_2017_9212_MOESM1_ESM.docx]

# Supplemental Results

| **Table S1.** Score distributions according to ASD affectation status | | | | | |  | |  | |  |  |
| --- | --- | --- | --- | --- | --- | --- | --- | --- | --- | --- | --- |
|  |  | **Unaffected siblings (1)** | | **Affected siblings (2)** | | **Probands (3)** | | | **F (*p*)** | | **Post-hoc** |
|  |  | **n** | **Mean (SD)** | **n** | **Mean (SD)** | **n** | **Mean (SD)** | |  |  |  |
| SRS-2 score | |  |  |  |  |  |  | |  | |  |
|  | Parent | 85 | 46.0 (7.0) | 28 | 74.6 (12.6) | 107 | 75.7 (12.3) | | 200.9 (<0.001) | | 1<2, 1<3 |
|  | Teacher | 80 | 49.1 (10.3) | 27 | 73.0 (12.5) | 107 | 68.3 (10.8) | | 89.3 (<0.001) | | 1<2, 1<3 |
| CBCL/TRF ADHP score | |  |  |  |  |  |  | |  | |  |
|  | Parent | 85 | 54.0 (6.8) | 29 | 61.3 (8,9) | 114 | 62.0 (9.4) | | 23.3 (<0.001) | | 1<2, 1<3 |
|  | Teacher | 79 | 52.9 (5.4) | 27 | 62.6 (8.5) | 113 | 60,3 (7,7) | | 31.7 (<0.001) | | 1<2, 1<3 |
| DCDQ score | |  |  |  |  |  |  | |  | |  |
|  | Parent | 24 | 68.1 (10.5) | 15 | 47.7 (15.0) | 44 | 44.2 (14.0) | | 25.9 (<0.001) | | 1>2, 1>3 |
| *Note.* SRS-2: Social Responsiveness Scale-2, T-score; CBCL/TRF ADHP: DSM-oriented Attention Deficit/Hyperactivity Problems Scale, T-score, from Child Behavior Checklist & Teacher Report Form; DCDQ: Developmental Disorder Coordination Questionnaire, adjusted total score. For SRS-2, CBCL, and TRF a higher score indicates more severe impairment. For DCDQ a higher score represents better functioning. For the SRS-2, a T-score of 30 is the minimum obtainable. A total T-score of 76 or higher is consistent with severe clinical-level symptomatology, a T-score of 60 through 75 subclinical, and a T-score of 59 or less as normal. For the CBCL and TRF, a T-score of 50 is the minimum obtainable. A T-score between 65 and 70 is considered borderline clinical and a score above 70 as clinical. | | | | | | | | | | | |

| **Table S2.** Intraclass correlations in ASD discordant and concordant sibling pairs | | | | | |
| --- | --- | --- | --- | --- | --- |
|  |  | **Discordant pairs (*n*=85)** | | **Concordant pairs (*n*=29)** | |
|  |  | ***n*** | **ICC (*p*)** | ***n*** | **ICC (*p*)** |
| SRS-2 score | |  |  |  |  |
|  | Parent | 78 | 0.10 (0.004) | 28 | 0.64 (0.005) |
|  | Teacher | 74 | 0.08 (0.165) | 27 | 0.77 (<0.001) |
| CBCL/TRF ADHP score | |  |  |  |  |
|  | Parent | 85 | 0.12 (0.201) | 29 | 0.46 (0.060) |
|  | Teacher | 78 | -0.28 (0.950) | 27 | 0.62 (0.009) |
| DCDQ score | |  |  |  |  |
|  | Parent | 19 | 0.04 (0.382) | 13 | -0.07 (0.545) |
| *Note.* SRS-2: Social Responsiveness Scale-2, T-score; CBCL/TRF ADHP: DSM-oriented Attention Deficit/Hyperactivity Problems Scale, T-score, from Child Behavior Checklist & Teacher Report Form; DCDQ: Developmental Disorder Coordination Questionnaire, adjusted total score. | | | | | |

| **Table S3.** Logistic regression analyses predicting sibling diagnosis, including both teacher- and parent-reported ADHD measures | | | | | | | | | | | |
| --- | --- | --- | --- | --- | --- | --- | --- | --- | --- | --- | --- |
|  | **Model 1** | |  | **Model 2** | |  | **Model 3** | |  | **Model 4** | |
|  | **n=35** | |  | **n=35** | |  | **n=35** | |  | **n=35** | |
|  | **OR** | **p** |  | **OR** | **p** |  | **OR** | **p** |  | **OR** | **p** |
| **Proband SRS-2 score** | 1.02 | 0.461 |  | 1.02 | 0.609 |  | 1.00 | 0.920 |  | 1.00 | 0.826 |
| **(teacher-report)** |  |  |  |  |  |  |  |  |  |  |  |
| **Sibling TRF ADHP score** |  |  |  | 1.15 | 0.033 |  | 1.13 | 0.021 |  | 1.11 | 0.074 |
| **(teacher-report)** |  |  |  |  |  |  |  |  |  |  |  |
| **Sibling CBCL ADHP score** |  |  |  |  |  |  | 1.20 | 0.006 |  | 1.21 | 0.020 |
| **(parent-report)** |  |  |  |  |  |  |  |  |  |  |  |
| **Sibling DCDQ score** |  |  |  |  |  |  |  |  |  | 0.92 | 0.041 |
| **(parent-report)** |  |  |  |  |  |  |  |  |  |  |  |
| **Nagelkerke R^2^** | 0.022 | |  | 0.283 | |  | 0.609 | |  | 0.722 | |
| *Note.* n=35 for all models (only siblings with all data available were included). SRS-2: Social Responsiveness Scale-2, T-score; TRF ADHP: DSM-oriented Attention Deficit/Hyperactivity Problems Scale, T-score, from Teacher Report Form; CBCL ADHP: DSM-oriented Attention Deficit/Hyperactivity Problems Scale, T-score, from Child Behavior Checklist; DCDQ: Developmental Disorder Coordination Questionnaire, adjusted total score. For SRS-2, TRF, and CBCL a higher score indicates more severe impairment. For DCDQ a higher score represents better functioning. | | | | | | | | | | | |

| **Table S4.** Reversed reporters: logistic regression analyses predicting sibling diagnosis | | | | | | | | | | | | | | |
| --- | --- | --- | --- | --- | --- | --- | --- | --- | --- | --- | --- | --- | --- | --- |
|  | **Model 1** | |  | **Model 2** | |  | **Model 3** | |  | **Model 4** | |  | **Model 5** | |
|  | **OR** | **p** |  | **OR** | **p** |  | **OR** | **p** |  | **OR** | **p** |  | **OR** | **p** |
| **Proband SRS-2 score (parent-report)** | 1.02 | 0.588 |  | 0.96 | 0.346 |  | 0.95 | 0.326 |  | 0.96 | 0.437 |  |  |  |
| **Sibling CBCL ADHP score (parent-report)** |  |  |  | 1.19 | 0.004 |  | 1.18 | 0.014 |  | 1.17 | 0.035 |  | 1.15 | 0.015 |
| **Sibling DCDQ score (parent-report)** |  |  |  |  |  |  | 0.91 | 0.014 |  | 0.88 | 0.016 |  | 0.91 | 0.017 |
| **CBCL ADHP x DCDQ interaction** |  |  |  |  |  |  |  |  |  | 1.01 | 0.130 |  |  |  |
| **Nagelkerke R^2^** | 0.012 | |  | 0.433 | |  | 0.637 | |  | 0.688 | |  | 0.616 | |
| *Note.* n=34 (only siblings with all data available). SRS-2: Social Responsiveness Scale-2, T-score; CBCL ADHP: DSM-oriented Attention Deficit/Hyperactivity Problems Scale, T-score, from Child Behavior Checklist; DCDQ: Developmental Disorder Coordination Questionnaire, adjusted total score. For SRS-2, and CBCL a higher score indicates more severe impairment. For DCDQ a higher score represents better functioning. | | | | | | | | | | | | | | |

| **Table S5.** Not restricted to siblings with all data: logistic regression analyses predicting sibling diagnosis | | | | | | | | | | | | | | |
| --- | --- | --- | --- | --- | --- | --- | --- | --- | --- | --- | --- | --- | --- | --- |
|  | **Model 1** | |  | **Model 2** | |  | **Model 3** | |  | **Model 4** | |  | **Model 5** | |
|  | **n=106** | |  | **n=99** | |  | **n=35** | |  | **n=35** | |  | **n=36** | |
|  | **OR** | **p** |  | **OR** | **p** |  | **OR** | **p** |  | **OR** | **p** |  | **OR** | **p** |
| **Proband SRS-2 score (teacher-report)** | 1.03 | 0.218 |  | 1.03 | 0.276 |  | 1.02 | 0.678 |  | 1.02 | 0.678 |  |  |  |
| **Sibling TRF ADHP score (teacher-report)** |  |  |  | 1.21 | <0.001 |  | 1.09 | 0.144 |  | 1.09 | 0.159 |  | 1.10 | 0.116 |
| **Sibling DCDQ score (parent-report)** |  |  |  |  |  |  | 0.92 | 0.010 |  | 0.92 | 0.013 |  | 0.92 | 0.010 |
| **TRF ADHP x DCDQ interaction** |  |  |  |  |  |  |  |  |  | 1.00 | 0.640 |  |  |  |
| **Nagelkerke R^2^** | 0.022 | |  | 0.370 | |  | 0.531 | |  | 0.537 | |  | 0.544 | |
| *Note.* SRS-2: Social Responsiveness Scale-2, T-score; TRF ADHP: DSM-oriented Attention Deficit/Hyperactivity Problems Scale, T-score, from Teacher Report Form; DCDQ: Developmental Disorder Coordination Questionnaire, adjusted total score. For SRS-2, and TRF a higher score indicates more severe impairment. For DCDQ a higher score represents better functioning. | | | | | | | | | | | | | | |

| **Table S6.** Specificity analyses: logistic regression analyses predicting sibling diagnosis | | | | | | | | | | | | | | |
| --- | --- | --- | --- | --- | --- | --- | --- | --- | --- | --- | --- | --- | --- | --- |
|  | **Model 1**  **n=99** | |  | **Model 1** | |  | **Model 2** | |  | **Model 3** | |  | **Model 4** | |
|  |  |  |  | **n=99** | |  | **n=99** | |  | **n=99** | |  | **n=99** | |
|  | **OR** | **p** |  | **OR** | **p** |  | **OR** | **p** |  | **OR** | **p** |  | **OR** | **p** |
| **Proband SRS-2 score (teacher-report)** | 1.03 | 0.276 |  | 1.02 | 0.513 |  | 1.03 | 0.201 |  | 1.04 | 0.132 |  | 1.02 | 0.505 |
| **Sibling TRF Attention Deficit/Hyperactivity Problems score (teacher-report)** | 1.21 | <0.001 |  |  |  |  |  |  |  |  |  |  | 1.20 | 0.003 |
| **Sibling TRF Affective Problems score (teacher-report)** |  |  |  | 1.23 | <0.001 |  |  |  |  |  |  |  | 1.21 | 0.009 |
| **Sibling TRF Anxiety Problems score (teacher-report)** |  |  |  |  |  |  | 1.02 | 0.603 |  |  |  |  | 0.89 | 0.020 |
| **Sibling TRF Oppositional Defiant Problems score**  **(teacher-report)** |  |  |  |  |  |  |  |  |  | 1.14 | 0.003 |  | 1.02 | 0.693 |
| **Nagelkerke R^2^** | 0.370 |  |  | 0.283 | |  | 0.031 | |  | 0.152 | |  | 0.485 | |
| *Note.* SRS-2: Social Responsiveness Scale-2, T-score; TRF: Teacher Report Form, DSM-oriented scales, T-score. For SRS-2, and TRF a higher score indicates more severe impairment. | | | | | | | | | | | | | | |

| **Table S7.** Linear regression analyses predicting parent-reported autistic trait severity in siblings, including both teacher- and parent-reported ADHD measures | | | | | | | | | | | |
| --- | --- | --- | --- | --- | --- | --- | --- | --- | --- | --- | --- |
|  | **Model 1** | |  | **Model 2** | |  | **Model 3** | |  | **Model 4** | |
|  | **n=35** | |  | **n=35** | |  | **n=35** | |  | **n=35** | |
|  | **β** | **p** |  | **β** | **p** |  | **β** | **p** |  | **β** | **p** |
| **Proband SRS-2 score** | 0.30 | 0.086 |  | 0.26 | 0.098 |  | 0.14 | 0.225 |  | 0.13 | 0.195 |
| **(teacher-report)** |  |  |  |  |  |  |  |  |  |  |  |
| **Sibling TRF ADHP score** |  |  |  | 0.45 | 0.005 |  | 0.39 | 0.002 |  | 0.25 | 0.020 |
| **(teacher-report)** |  |  |  |  |  |  |  |  |  |  |  |
| **Sibling CBCL ADHP score** |  |  |  |  |  |  | 0.58 | <0.001 |  | 0.43 | <0.001 |
| **(parent-report)** |  |  |  |  |  |  |  |  |  |  |  |
| **Sibling DCDQ score** |  |  |  |  |  |  |  |  |  | -0.43 | <0.001 |
| **(parent-report)** |  |  |  |  |  |  |  |  |  |  |  |
| **Adjusted R^2^** | 0.059 | |  | 0.247 | |  | 0.567 | |  | 0.709 | |
| *Note.* n=35 for all models (only siblings with all data available were included). SRS-2: Social Responsiveness Scale-2, T-score; TRF ADHP: DSM-oriented Attention Deficit/Hyperactivity Problems Scale, T-score, from Teacher Report Form; CBCL ADHP: DSM-oriented Attention Deficit/Hyperactivity Problems Scale, T-score, from Child Behavior Checklist; DCDQ: Developmental Disorder Coordination Questionnaire, adjusted total score. For SRS-2, TRF, and CBCL a higher score indicates more severe impairment. For DCDQ a higher score represents better functioning. | | | | | | | | | | | |

| **Table S8.** Reversed reporters: linear regression analyses predicting teacher-reported autistic trait severity in siblings | | | | | | | | | | | | | | |
| --- | --- | --- | --- | --- | --- | --- | --- | --- | --- | --- | --- | --- | --- | --- |
|  | **Model 1** | |  | **Model 2** | |  | **Model 3** | |  | **Model 4** | |  | **Model 5** | |
|  | **β** | **p** |  | **β** | **p** |  | **β** | **p** |  | **β** | **p** |  | **β** | **p** |
| **Proband SRS-2 score (parent-report)** | 0.22 | 0.203 |  | 0.06 | 0.735 |  | 0.07 | 0.684 |  | 0.10 | 0.526 |  |  |  |
| **Sibling CBCL ADHP score (parent-report)** |  |  |  | 0.44 | 0.016 |  | 0.28 | 0.119 |  | 0.26 | 0.143 |  | 0.30 | 0.067 |
| **Sibling DCDQ score (parent-report)** |  |  |  |  |  |  | -0.40 | 0.018 |  | -0.47 | 0.010 |  | -0.40 | 0.017 |
| **CBCL ADHP x DCDQ interaction** |  |  |  |  |  |  |  |  |  | 0.18 | 0.273 |  |  |  |
| **Adjusted R^2^** | 0.020 | |  | 0.163 | |  | 0.285 | |  | 0.291 | |  | 0.304 | |
| *Note.* n=34 (only siblings with all data available). SRS-2: Social Responsiveness Scale-2, T-score; CBCL ADHP: DSM-oriented Attention Deficit/Hyperactivity Problems Scale, T-score, from Child Behavior Checklist; DCDQ: Developmental Disorder Coordination Questionnaire, adjusted total score. For SRS-2, and CBCL a higher score indicates more severe impairment. For DCDQ a higher score represents better functioning. | | | | | | | | | | | | | | |

| **Table S9.** Not restricted to siblings with all data: linear regression analyses predicting parent-reported autistic trait severity in siblings | | | | | | | | | | | | | | |
| --- | --- | --- | --- | --- | --- | --- | --- | --- | --- | --- | --- | --- | --- | --- |
|  | **Model 1** | |  | **Model 2** | |  | **Model 3** | |  | **Model 4** | |  | **Model 5** | |
|  | **n=106** | |  | **n=99** | |  | **n=35** | |  | **n=35** | |  | **n=36** | |
|  | **β** | **p** |  | **β** | **p** |  | **β** | **p** |  | **β** | **p** |  | **β** | **p** |
| **Proband SRS-2 score (teacher-report)** | 0.21 | 0.032 |  | 0.17 | 0.044 |  | 0.19 | 0.109 |  | 0.19 | 0.111 |  |  |  |
| **Sibling TRF ADHP score (teacher-report)** |  |  |  | 0.53 | <0.001 |  | 0.24 | 0.066 |  | 0.25 | 0.074 |  | 0.23 | 0.074 |
| **Sibling DCDQ score (parent-report)** |  |  |  |  |  |  | -0.60 | <0.001 |  | -0.60 | <0.001 |  | -0.62 | <0.001 |
| **TRF ADHP x DCDQ interaction** |  |  |  |  |  |  |  |  |  | 0.03 | 0.793 |  |  |  |
| **Adjusted R^2^** | 0.034 | |  | 0.307 | |  | 0.554 | |  | 0.540 | |  | 0.525 | |
| *Note.* SRS-2: Social Responsiveness Scale-2, T-score; TRF ADHP: DSM-oriented Attention Deficit/Hyperactivity Problems Scale, T-score, from Teacher Report Form; DCDQ: Developmental Disorder Coordination Questionnaire, adjusted total score. For SRS-2, and TRF a higher score indicates more severe impairment. For DCDQ a higher score represents better functioning. | | | | | | | | | | | | | | |

| **Table**  **S10.** Unaffected siblings only: linear regression analyses predicting parent-reported autistic trait severity | | | | | | | | | | | | | | |
| --- | --- | --- | --- | --- | --- | --- | --- | --- | --- | --- | --- | --- | --- | --- |
|  | **Model 1** | |  | **Model 2** | |  | **Model 3** | |  | **Model 4** | |  | **Model 5** | |
|  | **n=79** | |  | **n=79** | |  | **n=74** | |  | **n=24** | |  | **n=23** | |
|  | **β** | **p** |  | **β** | **p** |  | **β** | **p** |  | **β** | **p** |  | **β** | **p** |
| **Proband SRS-2 score (teacher-report)** | 0.06 | 0.615 |  | 0.04 | 0.733 |  | 0.04 | 0.731 |  | -0.09 | 0.622 |  | -0.06 | 0.760 |
| **Sibling CBCL ADHP score (parent-report)** |  |  |  | 0.37 | 0.001 |  |  |  |  | 0.36 | 0.060 |  |  |  |
| **Sibling TRF ADHP score (teacher-report)** |  |  |  |  |  |  | 0.12 | 0.328 |  |  |  |  | -0.14 | 0.478 |
| **Sibling DCDQ score (parent-report)** |  |  |  |  |  |  |  |  |  | -0.46 | 0.018 |  | -0.61 | 0.004 |
| **Adjusted R^2^** | 0.000 | |  | 0.115 | |  | 0.000 | |  | 0.310 | |  | 0.261 | |
| *Note.* SRS-2: Social Responsiveness Scale-2, T-score; CBCL/TRF ADHP: DSM-oriented Attention Deficit/Hyperactivity Problems Scale, T-score, from Child Behavior Checklist/Teacher Report Form; DCDQ: Developmental Disorder Coordination Questionnaire, adjusted total score. For SRS-2, and CBCL/TRF a higher score indicates more severe impairment. For DCDQ a higher score represents better functioning. | | | | | | | | | | | | | | |

| **Table S11.** Specificity analyses: linear regression analyses predicting parent-reported autistic trait severity in siblings | | | | | | | | | | | | | | |
| --- | --- | --- | --- | --- | --- | --- | --- | --- | --- | --- | --- | --- | --- | --- |
|  | **Model 1** | |  | **Model 2** | |  | **Model 3** | |  | **Model 4** | |  | **Model 5** | |
|  | **n=99** | |  | **n=99** | |  | **n=99** | |  | **n=99** | |  | **n=99** | |
|  | **β** | **p** |  | **β** | **p** |  | **β** | **p** |  | **β** | **p** |  | **β** | **p** |
| **Proband SRS-2 score (teacher-report)** | 0.17 | 0.044 |  | 0.14 | 0.123 |  | 0.21 | 0.042 |  | 0.23 | 0.017 |  | 0.15 | 0.064 |
| **Sibling TRF Attention Deficit/Hyperactivity Problems score (teacher-report)** | 0.53 | <0.001 |  |  |  |  |  |  |  |  |  |  | 0.39 | <0.001 |
| **Sibling TRF Affective Problems score (teacher-report)** |  |  |  | 0.48 | <0.001 |  |  |  |  |  |  |  | 0.31 | 0.003 |
| **Sibling TRF Anxiety Problems score (teacher-report)** |  |  |  |  |  |  | 0.11 | 0.281 |  |  |  |  | -0.18 | 0.047 |
| **Sibling TRF Oppositional Defiant Problems score**  **(teacher-report)** |  |  |  |  |  |  |  |  |  | 0.35 | <0.001 |  | 0.08 | 0.373 |
| **Adjusted R^2^** | 0.307 | |  | 0.254 | |  | 0.038 | |  | 0.153 | |  | 0.371 | |
| *Note.* SRS-2: Social Responsiveness Scale-2, T-score; TRF ADHP: DSM-oriented Attention Deficit/Hyperactivity Problems Scale, T-score, from Teacher Report Form; DCDQ: Developmental Disorder Coordination Questionnaire, adjusted total score. For SRS-2, and TRF a higher score indicates more severe impairment. For DCDQ a higher score represents better functioning. | | | | | | | | | | | | | | |
